# Magnetic-Guided Axillary UltraSound (MagUS) Sentinel Lymph Node Biopsy and Mapping in Patients with Early Breast Cancer. A Phase 2, Single-Arm Prospective Clinical Trial

**DOI:** 10.3390/cancers13174285

**Published:** 2021-08-25

**Authors:** Allan Jazrawi, Eirini Pantiora, Shahin Abdsaleh, Daniel Vasiliu Bacovia, Staffan Eriksson, Henrik Leonhardt, Fredrik Wärnberg, Andreas Karakatsanis

**Affiliations:** 1 Centre for Clinical Research, County Västmanland, Uppsala University, 72189 Västerås, Sweden; allan.jazrawi@regionvastmanland.se (A.J.); staffan.eriksson@regionvastmanland.se (S.E.); 2 Department of Surgery, Västmanlands County Hospital, 72189 Västerås, Sweden; 3 Department of Surgical Sciences, Uppsala University, 75185 Uppsala, Sweden; eirini.pantiora@akademiska.se (E.P.); shahin.abdsaleh@akademiska.se (S.A.); fredrik.warnberg@vgregion.se (F.W.); 4 Department of Surgery, Section for Endocrine and Breast Surgery, Uppsala University Hospital, 75185 Uppsala, Sweden; 5 Aleris Mammography Unit, 75320 Uppsala, Sweden; 6 Department Immunology, Genetics and Pathology, Uppsala University, 75185 Uppsala, Sweden; daniel.vasiliu-bacovia@igp.uu.se; 7 Department of Radiology, Institute of Clinical Sciences, Sahlgrenska Academy, University of Gothenburg, 41343 Gothenburg, Sweden; Henrik.leonhardt@vgregion.se; 8 Department of Surgery, Institute of Clinical Sciences, Sahlgrenska Academy, University of Gothenburg, 41345 Gothenburg, Sweden

**Keywords:** sentinel lymph node biopsy, breast cancer, superparamagnetic iron oxide, magnetic tracer, sentinel lymph node

## Abstract

**Simple Summary:**

Superparamagnetic iron oxide nanoparticles (SPIO) have been shown to identify sentinel lymph nodes (SLNs) in patients with breast cancer. This study investigated whether a minimally invasive approach with MRI-LG after SPIO injection in the breast followed by a magnetic guided axillary ultrasound and core biopsy of the SLN (MagUS) could accurately stage the axilla. The study included not only patients planned for primary surgery but also patients with recurrent cancer after previous surgery, but also patients scheduled for neoadjuvant treatment (NAT). The latter underwent minimally invasive SLNB prior to treatment and had their SLN clipped; surgery in the axilla was performed after NAT. In 79 included patients, MagUS detected all patients with macrometastasis and performed comparably with surgical sentinel lymph node dissection (SLND). It also allowed for marking of the SLN in patients planned for PST and enabled tailored decision making in breast cancer recurrence.

**Abstract:**

Lymph Node Dissection (SLND) is standard of care for diagnosing sentinel lymph node (SLN) status in patients with early breast cancer. Study aim was to determine whether the combination of Superparamagnetic iron oxide nanoparticles (SPIO) MRI-lymphography (MRI-LG) and a Magnetic-guided Axillary UltraSound (MagUS) with biopsy can allow for minimally invasive, axillary evaluation to de-escalate surgery. Patients were injected with 2 mL of SPIO and underwent MRI-LG for SN mapping. Thereafter MagUS and core needle biopsy (CNB) were performed. Patients planned for neoadjuvant treatment, the SLN was clipped and SLND was performed after neoadjuvant with the addition of isotope. During surgery, SLNs were controlled for signs of previous biopsy or clip. The primary endpoint was MagUS SLN detection rate, defined as successful SLN detection of at least one SLN of those retrieved in SLND. In 79 patients, 48 underwent upfront surgery, 12 received neoadjuvant and 19 had recurrent cancer. MagUS traced the SLN in all upfront and neoadjuvant cases, detecting all patients with macrometastases (*n* = 10). MagUS missed only one micrometastasis, outperforming baseline axillary ultrasound AUS (AUC: 0.950 vs. 0.508, *p* < 0.001) and showing no discordance to SLND (*p* = 1.000). MagUS provides the niche for minimally invasive axillary mapping that can reduce diagnostic surgery.

## 1. Introduction

Primary tumor biology and axillary status guide therapeutic decisions in breast cancer treatment [1,2]. Sentinel Lymph Node Dissection (SLND) is considered the standard method of axillary staging, both in upfront surgery as well as after neoadjuvant treatment (NAT) [3,4,5,6,7,8].

Preoperative identification of patients with a negative SLN, or low-volume axillary disease that does not warrant further surgery, but guides therapeutic decisions, may allow for tailored approaches avoiding upfront SLND [6,9,10]. In patients scheduled for NAT, identifying those with a true negative axilla, but also those with low-volume disease, as de-escalation of axillary surgery after conversion from cN1 to cN0, could be safely attempted. [7,11,12].

At the same time, SLND is not an indolent procedure and is related to complications and considerable short- and long-term morbidity [13,14,15,16]. Therefore, non- or minimally invasive modalities have been proposed in order to address this problem. All of them are based on the principle of injecting a contrast interstitially in the breast in the same manner as when SLND is performed. The contrast will then be taken up by the lymphatics and reach the SLNs and will subsequently be visualized by a radiological modality. Previously, several methods such as single-photon emission computed tomography (SPECT), tridimensional computed tomography lymphography (3D-CTLG) or contrast enhanced ultrasound with microbubbles (CEUS) have been evaluated as alternatives to surgery [17,18,19]. Most of these have shown promising results, but larger studies are missing and, complicated logistics, need for access to nuclear medicine facilities and demanding learning curves are restricting their introduction into clinical practice.

Superparamagnetic iron oxide nanoparticles (SPIO) are used as a SLND tracer with comparable detection to the combination of radioisotope and blue dye, as shown in previous studies [20,21]. Additionally, when SPIO is injected in the breast, it can identify SLNs in axillary magnetic resonance imaging lymphography (MRI-LG) [22]. At the same time, SPIO yields the benefit that it resides in the tissue for a prolonged period of time without migrating to higher lymph node echelons and, thus, allows for the identification for SLNs during a much wider timeframe [23]. In this manner the SLNs that are identified during surgery should be visible in an MRI and, at the same time, transcutaneous signal detected by a magnetic probe, as in surgery, should be able to guide the axillary ultrasound to allow for transcutaneous identification and biopsy of the SLNs. Such a concept would have the perceived advantages of combining and tailoring modalities and at the same time, allowing for preoperative work up in a timeframe wider than the short halftime of Tc^99^ used for SPECT or that in the case of CEUS [19,24].

The development of an integrated technique bridging non-invasive and minimally invasive procedures for enhancement of the standard, axillary ultrasound-based diagnostic work-up is highly relevant [23,24,25]. The aim of this study was to determine whether the preoperative work-up with SPIO MRI-LG and Magnetic-guided Axillary UltraSound (MagUS), can accurately localize SLNs and predict SLN status and whether such a technique has the potential of replacing SLN surgery in the future.

## 2. Methods

### 2.1. Patients

Adult patients with clinically and ultrasound node-negative early breast cancer (cN0) planned for SLND at Uppsala University Hospital, from September 2017 to December 2020, were enrolled in the study after written informed consent. Patients with hypersensitivity to dextran compounds or SPIO, iron overload disease or planned for NAT and monitored with breast MRI for tumor response, were excluded. If a diagnostic breast MRI was needed, it was performed separately, before SPIO injection and axillary MRI-LG. The study was approved by the Regional Ethics Board in Uppsala (DNR 2016/385).

### 2.2. MRI-LG

Patients were injected peritumorally in the breast with 2 mL of SPIO (Magtrace^®^, Endomag., Cambridge, UK) and underwent MRI-LG one to 14 days after the injection. MRI-LG was performed with the patient in a supine position and adduction of the ipsilateral arm. The examination was performed without iv-contrast and took ca 8 min to complete. In cases of previous breast and axillary surgery or parasternal cancers, the contralateral axilla was also included in the MRI-LG to identify aberrant lymphatic outflow [26]. The MRI images were obtained using a 1,5-T and 3-T system (Philips^®^, Amsterdam, The Netherlands) with T2W cor, T2* tra and T2* cor sequences. Any lymph node with SPIO uptake in a T1 sequence or SPIO related void artifact on T2 sequence was considered a SLN, as previously described imaging was reviewed and the number of identified SLNs was documented [22]. SLN localization was described according to the classification proposed by Clough et al. [27], in relation to the lateral thoracic vein and the second intercostobrachial nerve. SLN metastatic status was assessed according to criteria previously proposed by Motomura et al. [22]; a lymph node was considered non-metastatic if there was a homogenous low intensity signal uptake of SPIO and metastatic if the entire node or a focal area did not show low signal intensity uptake.

### 2.3. Magnetic Guided Axillary UltraSound (MagUS) and Core Needle Biopsy (CNB)

**Figure 1:**
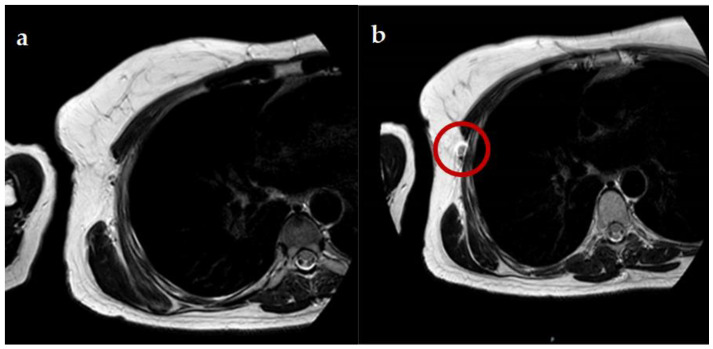
(**a**,**b**). Visualization of SLN with MRI before and after SPIO. In an enhancement of the SLN is visualized after injection of SPIO. The red circle visualizes the enhanced SLN after the injection of SPIO.

After reviewing of MRI-LG, the radiologist performed a second look axillary ultrasound in another session. The examination was focused to the area where the SLNs were identified on MRI (Figure 1). After a primary assessment for lymph nodes, a handheld magnetometer (Sentimag^®^, Endomag, Cambridge, UK) was used to identify the “pre-incision hotspot” which is the area with the highest magnetic uptake on the skin, and concordance with the MRI localization was registered.

**Figure 2:**
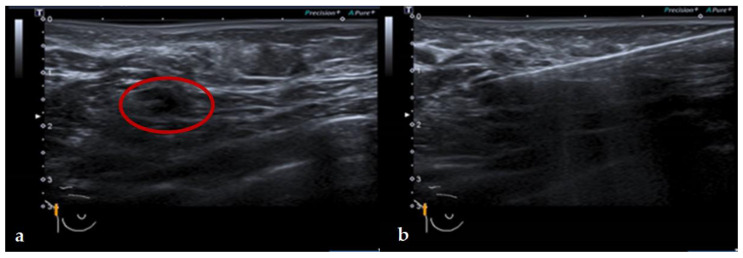
(**a**,**b**). MagUS with the SLN visualized in the red circle (**left**). Magnetic probe localizes the magnetic “hotspot” and after that CNB is performed (**right**). Monitor width 3.9 cm.

**Figure 3:**
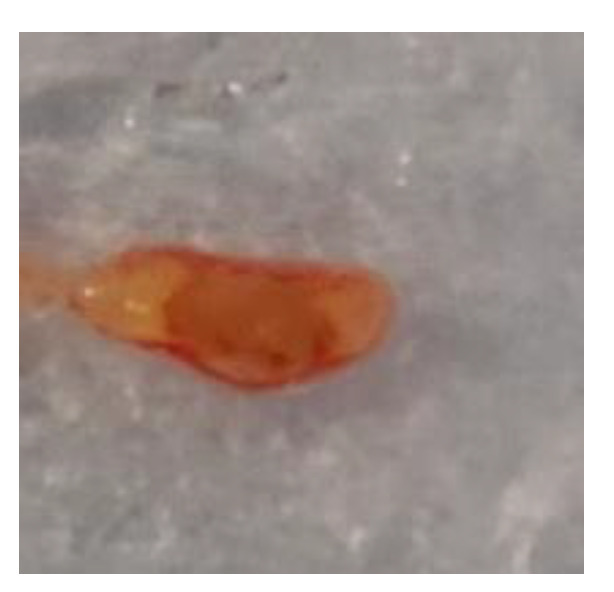
MagUS SLN-biopsy specimen (size 1 cm).

Subsequently, the identified lymph node(s) were assessed, and the percutaneous CNB of the SLN was performed with ultrasound guidance under local anesthesia (Figure 2). The CNB was evaluated for the presence of brown staining and magnetic uptake with the SentiMag probe (Figure 3). If more than one pathological lymph nodes were identified at this stage, the protocol stated that multiple efforts could be performed only after patient consent; otherwise, if the bioptic material obtained was considered representative and adequate, only the most prominent node was biopsied. Standard histopathologic analyses to assess metastasis was also performed, including verification of SPIO presence in the SLN. In patients undergoing NAT, the SLN was clipped simultaneously after the CNB, at the same session. When CNB was completed, the area was scanned for bleeding.

The study protocol ruled that the first five patients would undergo axillary MRI-LG before and after SPIO administration, and that MagUS and CNB was performed in the operation theatre, after the induction of anesthesia and right before surgery. In cases of recurrent breast cancer with aberrant SLN localization on MRI-LG and MagUS, a decision to attempt SLND was made at the multidisciplinary conference and after discussion with the patient. In patients undergoing NAT, a new axillary MRI-LG was performed after NAT, with no subsequent SPIO injection to see whether SPIO uptake in the SLNs was still visible. The number and localization of SLNs on MRI images was documented and axillary transcutaneous SentiMag signal was recorded. During subsequent SLND, concomitant radioisotope injection was administered and during surgery we registered which SLNs were magnetic, radioactive or both as well as the signal of the clipped node with both tracers.

### 2.4. Surgery and Specimen Pathology

During surgery, SLND was performed and the retrieved SLNDs were controlled macroscopically and microscopically for signs of previous biopsy, hematoma or the presence of clip, if placed. Standard pathology of the SLN specimen served as a reference to the microscopical examination of the CNB. 

**Figure 4:**
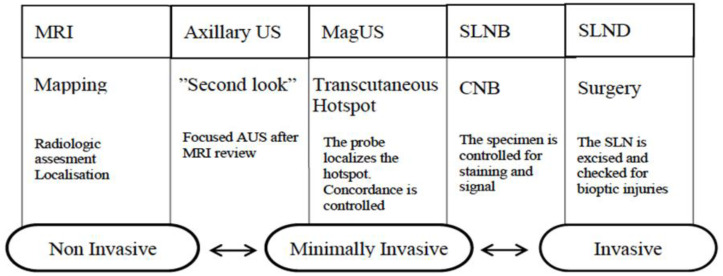
Flowchart showing the MagUS process.

The entire MagUs flowchart is summarized in Figure 4.

### 2.5. Trial Design and Study Endpoints

To assess whether the MagUS concept has the niche to replace surgical axillary evaluation (SLND), it was necessary to ensure concordance and agreement across the different modalities. With other words, it was necessary to verify that the SLNs identified and retrieved during surgery, were the same lymph nodes visualized on the MRI and the same that were detected by the magnetic probe, identified by the ultrasound and subsequently biopsied with a core needle. The common denominator was the presence of SPIO in the node and how this is demonstrated throughout the different modalities (MRI, MagUS, Surgery). Therefore, the outcome of interest was a minimum agreement in the assessment obtained by the MRI/MagUS with the standard of care, that is surgery. For this, it was clinically relevant to assess if the technique at hand is feasible, before venturing on a large clinical trial. Subsequently, the MagUS trial was conceived as a single stage phase 2 trial following the A’Hern’s design [28]. For a one-sided test a type one error a = 0.025 and 80% power, a sample size of 75 or more was required between a maximum futility proportion of 95% (corresponding to the proportion of successful detection above which the method can be further considered) and a minimum efficacy of proportion of 85% (corresponding to the proportion of successful detection under which, the method should not warrant further investigation).

The primary endpoint was determination of the MagUS SLN detection rate, defined as successful SLN detection of at least one SLN of those retrieved in the following SLND. Secondary endpoints were false-negative rate (FNR) of the MagUS technique, defined as no diagnosis of SLN metastasis (index test = negative) but presence of metastases by histopathology in any of the retrieved SLNs (reference test = positive) and overall accuracy, sensitivity, specificity and positive and negative predictive value (PPV, NPV).

Another aim of the study was to determine whether the MagUS technique could improve preoperative workup accuracy. For this, discordance in axillary evaluation from baseline clinical and ultrasonographical assessment was assessed.

Subgroup analyses were carried out to review the role of each component of the MagUS technique (MRI-LG. MagUS and MagUS core biopsy) and their potential role in tailored axillary mapping and inform on a future phase 3 trial.

The manuscript was prepared according to the Standards for the Reporting of Diagnostic Accuracy Studies (STARD) statement [29]. Descriptive statistics were performed by means of median (range) for continuous variables. Subsequently, non-parametric tests were used for comparisons. The McNemar’s test was used for the assessment of discordance in paired observations. For diagnostic accuracy statistics, Receiver Operating Characteristics (ROC) curves were constructed and the area under the curve (AUC) is provided. Effect sizes are provided with 95% confidence intervals (95% CI). Data analyses were performed using SPSS (V 26.0. IBM Corp, Armonk, NY, USA) and Stata^®^, version 16 (StataCorp LP, College Station, TX, USA).

## 3. Results

**Figure 5:**
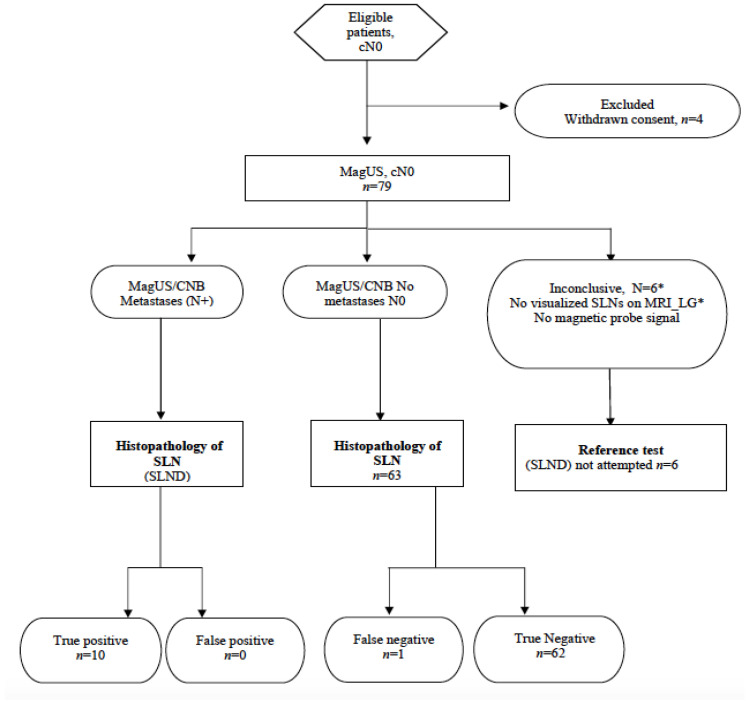
STARD flow diagram. * MRI_LG: Magnetic resonance imaging Lymphography. SLND: Sentinel Lymph Node Dissection.

The study is summarized in (Figure 5) and patient characteristics are presented in Table 1. In a total of 79 patients, 48 had early breast cancer and underwent upfront surgery, 12 underwent NAT and 19 had recurrent breast cancer after previous breast and axillary surgery.

MRI-LG was performed a median of 3 days after SPIO injection (range 1–12) and the MagUS with transcutaneous SLNB ± SLN clipping a median of 3 days (range 1–5) after MRI-LG. In all 73 patients where MagUS SLNB was performed, transcutaneous detection was successful and the SLN was located. Minimally invasive SLNB (MagUS CNB) retrieved lymphatic tissue with magnetic signal on the SentiMag^®^ probe, and the presence of SPIO was confirmed on post-operative histopathology. At surgery, the node with signs of previous biopsy and/or clip was always retrieved. In one case, the lymph node that was biopsied was a non-sentinel node (i.e., ex vivo signal less than 10% of the signal of the SLN with the maximal signal), but the true SLN was just behind it and recovered during SLND.

Metastases on specimen pathology was found in 11 patients (11/73, 15.1%, 95% confidence intervals: 7.8; 25.4). MagUS identified all patients with SLN macrometastases (*n* = 10) and missed only one SLN with a micrometastasis, resulting in a FNR of 8.3% and an overall accuracy of 98.6% (Table 2 and Table 3). In terms of diagnostic performance, when compared to the results of surgical pathology, MagUS performed very accurately (AUC: 0.955; 0.865, 1.000, *p* < 0.001) whereas AUS was not predictive at all (AUC: 0.505; 0.410, 0.601, *p* = 0.916)

The number of SLNs identified on MRI-LG (median 4, range 1–6) did not differ from the number of SLNs retrieved (median 3, range 1–6) (Wilcoxon signed rank test, *p* = 0.331) with high correlation (Cronbach’s Alpha = 0.719; 0.481, 0.848, *p* < 0.001). Additionally, topographic concordance between MRI-LG, MagUS and SLND was 100%. In 63 patients (86%), the nodes were located medial to the lateral thoracic vein and caudal to the intercostobrachial nerve.

In patients receiving NAT, the MagUS allowed for accurate axillary mapping, identification and clipping of the true SLN prior to the initiation of NAT. After the completion of NAT, a median of 130 days (range 86–140) after SPIO injection, the SLNs were still visualized in MRI-LG and were detectable during surgery in all patients. There was excellent correlation between the number of SLNs identified on MRI (median 4, range 2–6) and the magnetic SLNs retrieved (median 3.5, range 1–6) with Cronbach’s Alpha = 0.919; 0.699, 0.978, *p* < 0.001.

In patients with local recurrence after previous breast and axillary surgery (*n* = 19), MagUS showed either aberrant lymphatic outflow or no outflow in 9 patients (47.3%), preventing unnecessary ipsilateral axillary exploration. In the remaining 10 patients, both MagUS SLNB and subsequent surgery were successful.

## 4. Discussion

In this phase 2 trial, the MagUS technique (MRI-LG and MagUS) provided comparable results in accuracy and FNR with the standard of SLND. It was more accurate than the standard b-mode AUS in preoperatively detecting low-volume axillary disease. In this trial, it was demonstrated that accurate minimally invasive axillary staging can be achieved with a multimodal platform that can be modified to meet tailored patient needs.

SLND is not an indolent procedure and is related to short- and long-term morbidity such as postoperative pain, restricted shoulder range of motion, axillary web syndrome and lymphedema, as suggested in recent meta-analysis [13,14,30]. These findings indicate the need of establishing techniques for less invasive axillary staging that might result in less surgery, less subsequent postoperative complications and a reduction of costs and resources related with surgery [31,32]. Additionally, this MagUS workup can be performed in a wide timeframe and in an outpatient basis, as SPIO resides in the tissue a long period of time.

Recently, the necessity of surgical axillary mapping has been challenged in particular clinical scenarios. Observational data suggest that SLND may be safely omitted in older patients with primary tumors with small size and favorable biology [33,34,35]. The SOUND randomized trial examines whether a negative AUS can allow for the omission of SLND in patients with unifocal tumors < 2 cm planned for breast conservation and radiotherapy [36]. However, this approach does not take in consideration recent data that suggest that, in women with small tumors that are SLN negative, radiotherapy may be safely omitted nor that diagnosis of low-volume axillary disease, may allow for tailoring of radiotherapy or systemic treatment [6,9,37,38,39]. The results of the MagUS trial suggest that this technique may be used instead of SLND in selected cases.

It has been shown that 25% of patients considered as cN0 by AUS+/−FNAC will have a positive SLN in surgery. MagUS has the potential to correctly identify this low-volume axillary disease group, so that further treatment decisions may be tailored but without further axillary surgery, as it has been shown in landmark trials such as AMAROS, ACOSOG Z0011 or, more recently, the RxPonder trial [6,9,40]. Reversely, in women with one positive lymph node on standard AUS, MagUS could assess the volume of axillary disease in a more accurate manner. This is a group that often harbors a higher nodal disease burden [41]. However, other studies show that this is explained by the fact that the sensitivity of AUS + FNAC increases significantly in patients with higher risk for nodal metastasis [42]. At the same time, up to 43.2% of this patient group, will be found to have two or less metastatic nodes, meaning that ALND will have been overtreatment [10]. If MagUS shows that there is only low-volume axillary disease, then the patient may have the possibility to avoid overtreatment and tailor treatment decisions may be made after discussion in the multidisciplinary meeting [43].

Subsequently, MagUS may also address issues regarding axillary staging in the setting of NAT, as it yields the potential of differentiating patients that are clinically node negative from those who are also SLN negative prior to NAT. In this manner, therapeutic decisions regarding the axilla, such as axillary radiotherapy may be better tailored, while its definitive role in this setting remains still to be elucidated [44,45]. At the same time, it may answer whether, in cN positive patients, the metastatic node is a sentinel or if, at presentation, there are non-sentinel metastases, which is suggestive of a higher axillary nodal burden. In this manner, it becomes safer to identify more appropriate potential candidates for axillary conservation post-NAT as recently suggested in the Lucerne toolbox [12]. Moreover, MRI-LG before and after NAT allows for an estimate of the number of SLNs in the axilla. This may address the problem of FNR after NAT, that has been discussed in landmark trials, such as Sentina and ACOSOG Z1071 [46,47,48,49,50]. In these trials FNR was shown to decrease with the removal of ≥3 nodes, including clipped nodes, if such, whereas double tracer was shown to increase detection rate [7,46,47,48,49,50]. In the present study, post-NAT MRI-LG showed uptake in the same SLNs, suggesting that SPIO did not migrate in higher nodal echelons during NAT. Intraoperatively, there was transcutaneous magnetic signal and SLNs were detected in all cases. It may be so that, a MagUS could be repeated after NAT to allow for more focused axillary evaluation, as standard AUS has not shown promising results in this setting [51]. As omission of axillary surgery post neoadjuvant is discussed in several breast cancer subtypes, provided that there is pathologic complete response (PCR) in the breast, MagUS could provide a safer manner to discuss omission of surgery, rather than, in case of non-PCR, performing SLND that will be subject to the risk of false negatives post NAT and after a previous excision in the breast [52,53]. A given restriction is that SPIO injection in the breast impairs the diagnostic accuracy of the MRI, suggesting that the tumor response should be performed with other modalities. Reassuringly, modalities, such as ultrasound and PET-CT have shown comparable accuracy in this setting, without the known risk of false positive findings from the MRI [54,55,56,57].

Evaluating nodal status for breast cancer after previous breast and axillary surgery is a challenge. SLN detection rate is lower and aberrant, extra-axillary lymphatic drainage is not unusual [26,58,59]. For this reason, the use of preoperative mapping by means of scintigraphy is recommended in this setting. However, whilst accurate, scintigraphy complicates logistics and this is why it recent data suggest that it is no longer necessary for patients without previous breast or axillary surgery undergoing upfront SLND [60]. MagUS has, in this setting, allowed for tailored patient treatment with flexibility, as the MRI-LG performed preoperatively, allowed in good time to know whether SLND would be attempted on the day of surgery. In this manner, logistics were facilitated, and treatment decisions could be tailored with more precision and accuracy.

The strictly controlled study design allowed for safe results, despite the absence of a control arm. However, this is a phase 2 trial and these results need to be refined and reproduced in a larger scale. Consequently, a phase 3 randomized controlled trial is needed prior to standardization and routine adaptation of the technique instead of surgical SLND. The results suggest that MagUS has the potential to provide a substantial niche to avoid axillary surgery. The cost of surgery is the most substantial, especially if one takes the expenses related with leave of absence, morbidity and complication risks into consideration. Moreover, it is currently unclear whether the technique will always be implemented with the combination of an MRI and MagUS, something which might complicate and prolong the preoperative assessment of the patient. Finally if clinical MRI of the breast is intended, it should be performed first, to be followed by MagUs in another, different session. However, study results suggest that in women without risk factors for decreased ultrasound accuracy and transcutaneous magnetic probe detection (obesity, previous axillary surgery, etc.), MagUS and CNB were sufficient to accurately stage the axilla, suggesting that MRI is probably necessary in a small subgroup of patients (obesity, previous axillary surgery, etc.). This means that tailoring the technique to the specific patient will result in different routines and probably costs. Another substantial benefit is that this can be performed during the period between diagnosis a breast surgery, so that axillary mapping can be performed preoperatively and on an outpatient basis.

MagUS seems to be a method that can allow for alternatives to surgical axillary mapping. It comes to add to the armamentarium of other minimally invasive techniques that have previously been proposed [17,19,22,61] allowing for tailored axillary mapping in breast cancer. Its presumed advantages are the combination of different imaging modalities, together with that SPIO remains in the node a longer period, so as to allow for delayed SLND. Technique refinement and larger studies will allow for elucidation of the possibilities and its role in breast cancer diagnosis and treatment.

## 5. Conclusions

MagUS provides the niche for minimally invasive axillary mapping that can meet tailored patient needs and reduce diagnostic surgery. A phase 3 RCT is planned to further evaluate the technique.

## Figures and Tables

**Table 1:** Patient characteristics.

Patient Characteristics.	
Patient age at operation (median, range)	64 (38–87)
Body mass index (median, range)	24.8 (19.1–43.8)
Preoperative tumor extent mm (median, range)	20 (5–120)
Days between injection and Surgery (median, range)	12 (0–140)
Laterality, number, %	
Right	41 (51.9)
Left	38 (48.1)
Previous breast surgery	
Right	21 (26.6)
Left	58 (73.4)
Previous axillary surgery	
Right	19 (24.4)
Left	59 (75.6)
Neo adjuvant treatment	
Right	12 (15.2)
Left	67 (84.8)
Localization in the breast, number, %	
Upper outer	31 (39.2)
Upper inner	12 (15.2)
Lower outer	9 (11.4)
Lower inner	7 (8.9)
Central	7 (8.9)
Multicentric	11 (13.9)
Chest wall	2 (2.5)
Histological type (*n* = 79)	
Invasive ductal (*n*, (%))	66 (83.5)
Invasive lobular (*n*, (%))	11 (13.9)
Other Histology (*n*, (%))	2 (2.5)
Intrinsic Subtype (*n* = 79)	
Luminal A (*n*, (%))	36
Luminal B, erbb2− (*n*, (%))	20
Luminal B, erbb2+ (*n*, (%))	10
Non luminal erbb2+ (*n*, (%))	3
Triple negative (*n*, (%))	9
Type of surgery (*n* = 79)	
Wide local excision (*n*, (%))	28 (35.4)
Mastectomy (*n*, (%))	23 (29.1)
Oncoplastic breast conservation (*n*, (%))	28 (34.4)

**Table 2:** Comparison between MagUS and final pathology.

		Preoperative MagUS Assessment for Metastases		
		No *n*, (%)	Yes *n*, (%)	Total *n*, (%)
Metastases at histopathology	No	62 (98.4)	0 (0)	62 (84.9)
	Yes	1 (1.6)	10 (100)	11 (15.1)
Total		63 (100)	10 (100)	73 (100)

Mc Nemar’s test, *p* = 1.000.

**Table 3:** Diagnostic performance of the MagUS technique.

	Rate	Lower 95% CI	Upper 95% CI
Sensitivity	90.9%	58.7%	99.8%
Specificity	100%	94.2%	100%
PPV	100%	69.1%	100%
NPV	98.4%	91.5%	99.9%
Accuracy	98.6%	92.6%	99.9%

## Data Availability

The data presented in this study are available on request from the corresponding author. The data are not publicly available due to ethical considerations and data regulations.
